# A Unique Case of Valacyclovir Toxicity and Pseudobulbar Affect in a Patient On Peritoneal Dialysis

**DOI:** 10.7759/cureus.13494

**Published:** 2021-02-22

**Authors:** Waqas Memon, Emily K Rose, Ayesha Akram, Brian Simba

**Affiliations:** 1 Internal Medicine/Nephrology, Virginia Commonwealth University, Richmond, USA; 2 Internal Medicine, Virginia Commonwealth University, Richmond, USA; 3 Internal Medicine, Combined Military Hospital, Rawalpindi, PAK; 4 Internal Medicine, Rawalpindi Medical University, Rawalpindi, PAK

**Keywords:** peritoneal dialysis (pd), end stage renal disease (esrd), pseudobulbar affect, valacyclovir-induced neurotoxicity

## Abstract

There are a few cases of valacyclovir-associated neurotoxicity (VAN) reported. This case report documents a case of a 55-year-old male presenting with emotional lability or pseudobulbar affect as the predominant or sole manifestation of VAN. A failure to adjust valacyclovir’s dose for herpes simplex infection in the setting of dialysis-dependent end-stage renal disease (ESRD) preceded VAN in this patient. The patient presented with involuntary and uncontrollable outbursts of emotion. Computerized tomography (CT) scan identified no underlying cause. A complete neurological examination with cognitive assessment was performed, with no abnormalities. He benefited from the use of aggressive peritoneal dialysis (PD) that was employed to enhance valacyclovir’s clearance in this case of intractable VAN. On discharge, the patient was back to baseline mental function.

Traumatic brain injury, neoplasm, vascular lesions, metabolic abnormality, neurological disease, herpetic encephalitis, and disorders of mood were ruled out. This led to the hypothesis of encephalopathy due to valacyclovir intoxication. Given that the clinical manifestations were related to ESRD, a dose-adjustment of valacyclovir is imperative in the setting of ESRD to prevent VAN. Our case presents important clinical variations. Firstly, our patient demonstrates that VAN may present with no focal neurological impairment, but pseudobulbar affect. Secondly, aggressive PD was useful in this case for the treatment of VAN as opposed to hemodialysis. We believe that it cleared valacyclovir resulting in the resolution of symptoms.

## Introduction

Valacyclovir dosing in end-stage renal disease (ESRD) is important to adhere to, given the risk of valacyclovir neurotoxicity. This is a case of valacyclovir neurotoxicity in a patient on peritoneal dialysis (PD), presenting with pseudobulbar affect.

## Case presentation

A 55-year-old male with ESRD due to hypertensive nephrosclerosis on PD, diabetes mellitus type two, chronic anemia, and hypertension presented to the hospital with emotional lability for three days. Several days prior to the patient’s presentation, he was prescribed valacyclovir, for orolabial herpes. The patient’s wife noticed that her husband was more confused at home, with frequent emotional outbursts and frequent emotional liability. On presentation to the emergency room, he was confused about why he was crying and denied feelings of depression or suicidal ideation. Other than recent diarrhea, he denied any other symptoms. Of note, the patient did not have any history of physiatric illness, alcohol, or drug use. 

**Figure 1:**
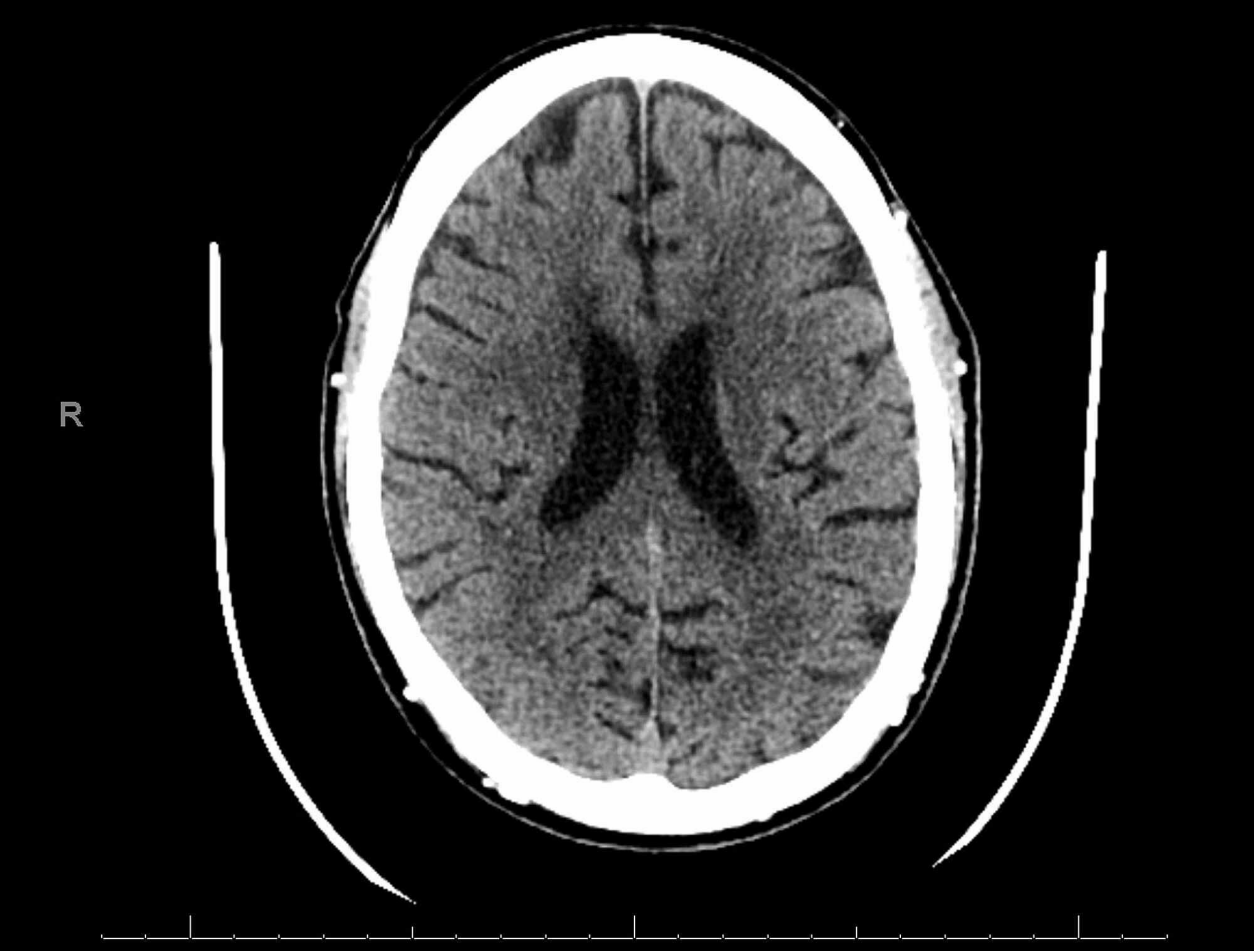
CT of the head revealing no acute intracranial hemorrhage, midline shift, mass effect, or extra-axial fluid collection. The gray-white differentiation is preserved. Brain volume and ventricular system are within normal limits for age

On physical exam, his vitals revealed a temperature of 36.7 °C, blood pressure of 158/97 mmHg, heart rate of 86 beats/min, and saturating at 96% on room air. He had no focal neurological deficits, or other concerning exam findings, though would cry intermittently during interviews. His labs showed sodium of 140 mmol/L, potassium 3.2 mmol/L, chloride 100 mmol/L, carbon dioxide 23 mmol/L, glucose 70 mg/dL, blood urea nitrogen (BUN) of 35 mg/dL, creatinine of 17.70 mg/dL, calcium of 6.4 mg/dL, magnesium of 1.4 mg/dL, and phosphorus of 9.8 mg/dL. White blood cell count of 8.6 10e9/L, red blood cell count of 3.93 10e12/L, hemoglobin of 11.6 g/dL, hematocrit of 34.4%. The patient also had an anion gap of 17. Blood and urine cultures were negative. The urine drug screen was also negative. The folic acid level was 10 ng/mL, B12 level was 650 pg/mL. Computerized tomography (CT) of the head showed no acute intracranial abnormality (Figure 1). There was no concern for infection or issues with PD. Nephrology was consulted to manage PD; however, after reviewing the history, it was discerned that he will need more frequent PD exchanges after recently being prescribed valacyclovir. After one day of increased PD exchanges, the patient’s emotional state improved significantly. 

## Discussion

Valacyclovir is a prodrug that undergoes first-pass metabolism in the gastrointestinal tract and the liver to acyclovir and L-valine. It is then eliminated renally, making dose adjustment necessary in patients with renal impairment [1]. After conversion to acyclovir, it has a half-life of 2.5-3.3 hours in adults with normal kidney function, which may increases to 14-20 hours in patients with ESRD [1]. Generally, 89% of the medication is excreted by the kidneys, explaining why a dose adjustment of valacyclovir is necessary for patients with renal impairment [1]. Acyclovir, valacyclovir, or famciclovir can treat primary oral herpes simplex virus (HSV) infection due to HSV-1, but valacyclovir is preferred due to better oral bioavailability and ease of once-daily dosing [2]. A short course of treatment with oral valacyclovir during a primary infection usually reduces the duration of viral shedding, time for lesional healing, and local pain.

Valacyclovir-associated neurotoxicity (VAN) is known to occur, though is still rare, and most often occurs in people with chronic renal disease or renal malfunction [3]. Cases of valacyclovir toxicity have typically reported that the toxicity occurs within 24 to 72 hours, and typical neurological symptoms include hallucinations, confusion, lethargy, fear of death, ataxia, and death [4]. Less common symptoms are dysarthria, myoclonus, and rhabdomyolysis [5]. These symptoms that have been reported occur in patients on either hemodialysis or PD and typically resolve two to seven days after removing the offending medication [5,6].

Typically, in situations with less severe valacyclovir toxicity, removal of the offending medication and supportive care are the primary treatment. However, in more severe cases, it has been reported many times that hemodialysis sessions enhance acyclovir elimination [7]. Stathoulopoulou et al. published a study investigating the pharmacokinetics of valacyclovir in patients on PD and found supratherapeutic levels of acyclovir in patients who received the recommended dose for patients without renal failure [8]. According to Asahi et al., hemodialysis enhances valacyclovir neurotoxicity recovery, but PD does not [4]. However, that statement was based on a review article by Adair et al. that discusses prompt removal of acyclovir via hemodialysis rather than PD, particularly when a patient’s neurological dysfunction is severe [9]. These authors also found that 24 hours after giving a dose of valacyclovir, 6.5 +/- 1.81% of acyclovir was excreted via the peritoneum [8]. This lends support as to why, in instances of severe toxicity, hemodialysis has been initiated to rapidly eliminate valacyclovir [10,11]. Nevertheless, the removal of acyclovir via PD has been done in the past. In reality, there are case reports that document improvement in neurological function after initiating a more aggressive PD regimen [5,6,9,10] (Table 1).

**Table 1:** The reported cases with valacyclovir-induced neurotoxicity in end-stage renal disease patients in the literature VAN: valacyclovir-induced neurotoxicity; PD: peritoneal dialysis.

Case:	Pipili et al. [6]	Prasad et al. [5]	Takayanagi et al. [12]	This case
Age/gender of the patient	72-year-old-female	57-year-old-female	67-year-old-male	55-year-old-male
Infection	Herpes zoster	Acute attack of herpes zoster	Herpes zoster	Orolabial herpes
Symptoms of VAN	Dysarthria, dysesthesia, impaired coordination, agitation, dizziness, visual hallucinations	Confusion, irritability, headaches, fine tremors, myoclonic jerks, ataxia	Fretful, hallucinations	Pseudobulbar affect
Dose of oral valacyclovir (unadjusted)	3 g/day	3 g/day	1 g/day	4 g/day
Recommended valacyclovir dose	500 mg/day	500 mg/day	500 mg/day	500 mg once
Initial PD dose	Three exchanges of 2 liters 2.5% dextrose plus one exchange of 2 liters icodextrin overnight	One nocturnal exchange of 7.5%, 1.5 Liter dianeal fluid	Not specified in the abstract	Four exchanges of 2 liters 2.5% dextrose
Intensification of PD regimen	Five exchanges of 2 liters 2.5% dextrose plus one exchange of 2 liters icodextrin overnight	Valacyclovir was held and she was continued on one exchange of 7.5%, 1.5 liters for 48 hours. Since there was no clinical improvement PD was intensified to six exchanges.	Continuation of initial PD without intensification	2.5% dextrose 2L exchanges every 3 hrs
Result of continuation/intensification of PD	Improvement in neurological status after 24 hours, with resolution 3 days later, and no recurrence at weekly follow-up	Remarkable improvement in neurological status after 24 hours, with discharge 5 days later	No neurological symptoms 7 days later	Pathological crying subsided after 24 hours

Izzedine et al., in another case, report of a single patient on continuous ambulatory peritoneal dialysis (CAPD) who developed VAN despite dose adjustment, found a CAPD clearance of only 5.27 mL/min [13]. Overall, the CAPD was able to remove less than 1% of the administered dose of the drug in 24 hours. However, after 48 hours of discontinuation of valacyclovir, the patient had recovered neurological status, proving PD is beneficial to some extent. Unfortunately, there are currently no randomized controlled trials that could guide and further allow us to reach a conclusion on the degree to which PD is effective.

This case demonstrates a unique presentation of a patient with emotional lability, without other abnormal neurological or psychiatric findings. In this particular case, the patient’s creatinine clearance was less than 10, so the recommended dose of valacyclovir for orolabial herpes is 500 mg one time. This patient instead received a total of 4 grams over a two-day period. After undergoing a more aggressive PD regimen, the patient’s emotional lability improved.

It is hypothesized that pseudobulbar affect is caused by disruption of neural networks that control the motor output of emotions, leading to activation of laughing or crying pathways in the brainstem even when the patient is not feeling that way on the inside, or by a decrease in the levels of monoamines like serotonin and dopamine in cortical limbic and cerebellar pathways [12]. However, the exact mechanism by which VAN occurs or pseudobulbar affect occurred in this patient remains largely speculative. Pre- and post-intensification acyclovir levels, and serum or cerebrospinal fluid (CSF) levels of the main acyclovir metabolite 9-carboxymethyl methylguanine (CMMG) were not determined, making this a slight limitation of this case. However, an entirely reasonable alternative approach is to rule out traumatic brain injury, neoplasm, vascular lesions, metabolic abnormality, neurological disease, herpetic encephalitis (as is evident by a normal CT scan, and the absence of systemic symptoms (e.g., fever) and focal neurological deficits on examination), and disorders of mood (there were no depressive symptoms, manic symptoms, delusions, and hallucinations) in a patient on valacyclovir. The patient was thus treated empirically for VAN by an intensification of PD, which proved beneficial. Since the patient was recently prescribed a new medication, and no other etiology was found to have caused the patient’s emotional lability, it is presumed that the valacyclovir likely caused neurotoxicity, resulting in the patient’s unique emotional lability, and the improvement via PD, which has been reported relatively infrequently [5,6,14].

## Conclusions

In summary, we present a unique case of emotional lability associated with valacyclovir neurotoxicity in a patient on PD, that improved with an aggressive PD regimen. Once the most likely etiology for neurologic or psychiatric dysfunction is thought to be due to valacyclovir, it is necessary to determine how to resolve the patient’s symptoms. In patients on PD in particular, it is important to know that unless a patient is severely neurologically impaired and needs emergent HD, that it is reasonable to try an aggressive PD regimen. This will avoid an unnecessary procedure and intervention for a patient and will allow a patient to undergo a familiar form of dialysis. Finally, the dosing of medications in patients with renal impairment cannot be emphasized enough. While easy to overlook, it should cross providers’ minds when prescribing any medication to a patient with chronic kidney or ESRD.
